# Diversity of *Trichoderma* spp. causing *Pleurotus* green mould diseases in Central Europe

**DOI:** 10.1007/s12223-012-0214-6

**Published:** 2012-11-29

**Authors:** Lidia Błaszczyk, Marek Siwulski, Krzysztof Sobieralski, Dorota Frużyńska-Jóźwiak

**Affiliations:** 1 Institute of Plant Genetics, Polish Academy of Sciences, Strzeszyńska 34, 60-479 Poznań, Poland; 2 Department of Vegetable Crops, University of Life Sciences, Dąbrowskiego 159, 60-594 Poznań, Poland; 3 Department of Phytopathology, University of Life Sciences, Dąbrowskiego 159, 60-594 Poznań, Poland

## Abstract

The present study includes the molecular characteristics of *Trichoderma pleurotum* and *Trichoderma pleuroticola* isolates collected from green moulded cereal straw substrates at 47 oyster mushroom farms in Poland. The screening of the 80 *Trichoderma* isolates was performed by morphological observation and by using the multiplex PCR assay. This approach enabled specific detection of 47 strains of *T. pleurotum* and 2 strains of *T. pleuroticola*. Initial identifications were confirmed by sequencing the fragment of internal transcribed spacer regions 1 and 2 (ITS1 and ITS2) of the rRNA gene cluster and the fragment including the fourth and fifth introns and the last long exon of the translation–elongation factor 1-alpha (*tef1*) gene. ITS and *tef1* sequence information was also used to establish the intra- and interspecies relationship of *T. pleurotum* and *T. pleuroticola* originating from the oyster mushroom farms in Poland and from other countries. Comparative analysis of the ITS sequences showed that all *T. pleurotum* isolates from Poland represent one haplotype, identical to that of *T. pleurotum* strains from Hungary and Romania. Sequence analysis of the *tef1* locus revealed two haplotypes (“T” and “N”) of Polish *T. pleurotum* isolates. The “T” type isolates of *T. pleurotum* were identical to those of strains from Hungary and Romania. The “N” type isolates possessed a unique *tef1* allele. Detailed analysis of the ITS and *tef1* sequences of two *T. pleuroticola* isolates showed their identicalness to Italian strain C.P.K. 1540.

## Introduction

*Pleurotus ostreatus* (Jacq.) P. Kumm. is one of the most important commercial crop edible mushrooms in Poland. Together with Italy and Hungary, Poland is the main producer of *P. ostreatus* in Europe. However, significant disintegration of oyster mushroom production and differences in cultivation conditions affect the appearance of many pests and diseases. In recent years, severe symptoms of green mould have been observed in oyster mushroom farms, resulting in crop losses.

The first reported appearance of green mould on *P. ostreatus* was in North America (Sharma and Vijay 1996). Serious cases of this disease in commercially grown *P. ostreatus* were detected thereafter in South Korea (Park et al. 2004a, b), Italy (Woo et al. 2004), Romania (Kredics et al. 2006), Hungary (Hatvani et al. 2007), and most recently in Spain (Gea 2009).

The causal agents of the *Pleurotus* green mould are two species of *Trichoderma*, which have been recently described as *Trichoderma pleurotum* S.H. Yu & M.S. Park and *Trichoderma pleuroticola* S.H. Yu & M.S. Park (Park et al. 2004a, b, 2006; Komon-Zelazowska et al. 2007). Phenotypically, *T. pleurotum* and *T. pleuroticola* species are significantly different. *T. pleuroticola* shows a typical pachybasium-like conidiophore developing in fascicles or pustules which is typical for the Harzianum clade, whereas *T. pleurotum* is characterised by a gliocladium-like conidiophore morphology (Park et al. 2006; Komon-Zelazowska et al. 2007). In spite of the large phenetic divergence, these species present a very close phylogenetic relationship to the Harzianum clade of *Hypocrea*/*Trichoderma*, which also includes *Trichoderma aggressivum* Samuels & W. Gams, the causative agent of green mould disease in *Agaricus* (Park et al. 2004a, b; Hatvani et al. 2007; Komon-Zelazowska et al. 2007).

*Trichoderma pleurotum* has been found only on cultivated *P. ostreatus* and its substratum. In contrast, *T. pleuroticola* has been found both on wild and cultivated *P. ostreatus*, as well as on the natural and productive substratum of the oyster mushroom (Park et al. 2004a, b; Szekeres et al. 2005; Hatvani et al. 2007; Komon-Zelazowska et al. 2007; Kredics et al. 2009). Additionally, *T. pleuroticola* has been isolated from soil and wood in Canada, the USA, Europe, Iran, and New Zealand (Park et al. 2004a, b; Szekeres et al. 2005; Komon-Zelazowska et al. 2007).

Until now, it has not been clear which species of *Trichoderma* is the causative agent of the green mould in oyster mushroom farms of Central Europe. The present study was carried out to confirm the association of the *T. pleurotum* and *T. pleuroticola* species with *P. ostreatus* cultivated in Poland based on morphological and molecular analysis of collected *Trichoderma* isolates originating from Polish oyster mushroom farms.

## Materials and methods

### Fungal collection

Four *T. pleurotum* (E135, E136, E138, E139) and five *T. pleuroticola* strains (E137, M141, M142, M143, M144), used as the reference strains, were kindly supplied by Dr. Monika Komon-Zelazowska, Research Area Gene Technology and Applied Biochemistry, Institute of Chemical Engineering, Vienna University of Technology, Austria. Eighty *Trichoderma* isolates were collected from green moulded cereal straw substrates at 47 oyster mushroom farms in Poland. The small fragments of cereal straw were taken from substrates used for cultivation of *P. ostreatus*. Basidiomes were suspended in 10 mL sterile distilled water and 0.2 mL Tween 20 (Sigma), incubated at 25 °C for 10 min on a rotary shaker (120 rpm) and diluted 1:10 with sterile distilled water. Inoculation was performed from the suspensions (0.5 mL) onto a potato dextrose agar (PDA, Oxoid) and incubated in darkness at 25 °C for 7 days. The resultant fungal colonies were transferred to new plates of PDA and incubated as described above. The strains collected from Polish mushroom farms and investigated in this study are listed in Table 1.

**Table 1:** The list of strains collected from oyster mushroom farms in Poland and identified on the basis of multiplex PCR and ITS and *tef1* sequence analysis

Culture code	Origin–localization		ITS and *tef1* sequence-based identification	
			Type of *tef1* allele^a^	
T24//T	Western Poland	Babimost	*T. pleuroticola*	–
TH/24		Babimost	*T. harzianum*	**–**
T72/A		Budziłowo	*T. pleurotum*	“T”
TV72/C		Budziłowo	*T. atroviride*	**–**
T63/DR		Chobienice	*T. pleurotum*	“N”
T14/Ł		Chrosnica	*T. pleurotum*	“T”
T58/2A		Kalisz	*T. pleurotum*	“N”
Tv57/2B		Kalisz	*T. atroviride*	**–**
TB112		Konin	*T. pleurotum*	“N”
T155		Łobez	*T. pleurotum*	“T”
TH55/F		Łobez	*T. harzianum*	**–**
TH25/A		Łobez	*T. harzianum*	**–**
TV55/Ł		Łobez	*T. atroviride*	**–**
T16/2/A^b^		Nądnia	*T. pleurotum*	“N”
T37/Ł		Nowy Tomyśl	*T. pleurotum*	“N”
TH370		Nowy Tomyśl	*T. harzianum*	**–**
Tv37/10		Nowy Tomyśl	*T. atroviride*	**–**
T71/B		Pleszew	*T. pleurotum*	“N”
T83/TB		Skoków	*T. pleurotum*	“N”
T12/B^b^		Widzim Stary	*T. pleuroticola*	**–**
TP53Ł		Wielichowo	*T. pleurotum*	“N”
Th530		Wielichowo	*T. harzianum*	**–**
T52/2D		Witaszyce	*T. pleurotum*	“N”
Tv52/G		Witaszyce	*T. atroviride*	**–**
TP81R		Wolsztyn	*T. pleurotum*	“N”
T2/DR		Wroniary	*T. pleurotum*	“T”
TP12/S	Northern Poland	Człopa	*T. pleurotum*	“T”
T05C		Gryfino	*T. pleurotum*	“T”
TB40/M		Jakubowo Kisielickie	*T. pleurotum*	“T”
T13/CB		Kamionki	*T. pleurotum*	“T”
Tv130/A		Kamionki	*T. atroviride*	**–**
T77/3		Kłębowo	*T. pleurotum*	“N”
TB63		Kłębowo	*T. pleurotum*	“T”
Tv76/D		Kłębowo	*T. atroviride*	**–**
Th76/K		Kłębowo	*T. harzianum*	**–**
T50A		Kołaczkowo	*T. pleurotum*	“N”
T36/Bi		Koszalin	*T. pleurotum*	“N”
Tv37/Ci		Koszalin	*T. atroviride*	**–**
T6/AR		Krępsko	*T. pleurotum*	“T”
TP32M		Kudypy	*T. pleurotum*	“N”
TH320		Kudyby	*T. harzianum*	**–**
T41/T		Opatów	*T. pleurotum*	“N”
TH410		Opatów	*T. harzianum*	**–**
TB18A		Przechlewo	*T. pleurotum*	“N”
TP19/S		Zblewo	*T. pleurotum*	“T”
Th19/B		Zblewo	*T. harzianum*	**–**
T270/C		Żodyń	*T. pleurotum*	“T”
Th271/B		Żodyń	*T. harzianum*	**–**
TP17M	Eastern Poland	Garbów	*T. pleurotum*	“T”
TH17/7		Garbów	*T. harzianum*	**–**
T53B		Grodzisk Mazowiecki	*T. pleurotum*	“N”
TP25S		Łosice	*T. pleurotum*	“N”
TB73/L		Nowa Huta	*T. pleurotum*	“N”
TB6M		Radom	*T. pleurotum*	“N”
Th6/M4		Radom	*T. harzianum*	**–**
TP20S		Siedlce	*T. pleurotum*	“N”
T27/Z		Siedlce	*T. pleurotum*	“N”
Tv18/AB		Siedlce	*T. atroviride*	**–**
TB27S		Wiśniew	*T. pleurotum*	“N”
T127		Wola Łaska	*T. pleurotum*	“N”
Tv127/RU		Wola Łaska	*T. atroviride*	**–**
T35A^b^	Southern Poland	Bratkowice	*T. pleurotum*	“T”
TB33		*Brzeźnica*	*T. pleurotum*	“T”
TV/33a		Brzeźnica	*T. atroviride*	**–**
TP11Ł		Bytom	*T. pleurotum*	“N”
T4/15/A		Czermin	*T. pleurotum*	“T”
TH15/C		Czermin	*T. harzianum*	**–**
TV17/C		Czermin	*T. atroviride*	**–**
T158/1		Ćwiklice	*T. pleurotum*	“T”
TH58/2		Ćwiklice	*T. harzianum*	**–**
TP23M		Kraków	*T. pleurotum*	“N”
TB2		Opole	*T. pleurotum*	“N”
Th2/33		Opole	*T. harzianum*	**–**
TB103		Pszczyna	*T. pleurotum*	“N”
Th10/PS		Pszczyna	*T. harzianum*	**–**
Th11/PS		Pszczyna	*T. harzianum*	**–**
Tv104/PS		Pszczyna	*T. atroviride*	**–**
T55Z/2		Ręczno	*T. pleurotum*	“T”
TB108		Smyków	*T. pleurotum*	“T”
Tv18/AB		Smyków	*T. atroviride*	**–**

^a^The type of allele observed on the basis of *tef1* sequence analysis of *T. pleurotum* isolates

^b^The representatives of *T. pleurotum* and *T. pleuroticola* isolates used in comparative analysis (Figs. 1 and 2)

### Morphological analysis

Identification was performed by observation of phenotypic characteristics of the colonies and by microscopic studies of the conidia and conidiophores. Colony characteristics were examined from cultures grown in darkness at 25 °C for 7 days on PDA. Microscopic observations were made according to Park et al. (2006).

### DNA isolation and amplification

Mycelium for DNA extraction was obtained as described previously (Błaszczyk et al. 2011). Isolation of total DNA was performed using the CTAB method (Doohan et al. 1998).

The ITS1 and ITS2 region of the rDNA gene cluster was amplified using primers ITS4 and ITS5 (White et al. 1990). A fragment of 1.2-kb *tef1* gene was amplified using primers Ef728M (Carbone and Kohn 1999) and TEF1LLErev (Jaklitsch et al. 2005) as well as the set of primers (FPforw1, FPrev1, PSrev1) designed for the rapid detection of *T. pleurotum* and *T. pleuroticola* (Kredics et al. 2009).

The PCR reaction was carried out in 25 μL reaction mixture containing: 1 μL 50 ng/μL of DNA, 2.5 μL 10 × PCR buffer (50 mmol/L KCl, 1.5 mmol/L MgCl_2_, 10 mmol/L Tris–HCl, pH 8.8, 0.1 % Triton X-100), 1.5 μL 10 mmol/L dNTP (GH Healtcare), 0.2 μL 100 mmol/L of each primer, 19.35 μL MQ H_2_O, 0.25 μL (2 U/μL) DyNAzymeTM II DNA Polymerase (Finnzymes) using a PTC-200 thermocycler (MJ-Research, USA). A multiplex PCR assay with *tef1* sequence-based primers FPforw1, FPrev1, PSrev1 was carried out under the conditions described by Kredics et al. (2009). Amplifications of ITS region and the fragment of *tef1* gene were performed as follows: initial denaturation 5 min at 94 °C, 35 cycles of 45 s at 94 °C, 45 s at 58 °C (for ITS region) or 63 °C (for *tef1* fragment), 1 min at 72 °C, with the final extension of 10 min at 72 °C.

Amplification products were separated on 1.5 % agarose gel (Invitrogen) in 1× TBE buffer (0.178 mol/L Tris-borate, 0.178 mol/L boric acid, 0.004 mol/L EDTA) containing ethidium bromide. A 100-bp DNA Ladder Plus (Fermentas) was used as a size standard. PCR products were electrophoresed at 3 V/cm for about 2 h, visualized under UV light, and photographed (Syngen UV visualiser).

### DNA sequencing and comparative analyses

The 0.4-kb ITS and 1.2-kb *tef1* amplicon purification steps and sequencing were carried out as described previously (Chełkowski et al. 2003; Błaszczyk et al. 2011). Sequences were edited and assembled using Chromas v. 1.43 (Applied Biosystems). The sequences were identified by BLASTn (http://blast.ncbi.nlm.nih.gov/) as well as *Trich*OKEY and *Tricho*BLAST (http://www.isth.info; Druzhinina et al. 2005; Kopchinskiy et al. 2005).

The comparative analyses were based on the ITS and *tef1* sequences of the 49 *T. pleurotum*/*T. pleuroticola* isolates obtained in the present study and 9 reference strains, as well as on the sequences of 21 other *T. pleurotum*/*T. pleuroticola* strains, deposited in NCBI GeneBank (www.ncbi.nlm.nih.gov, Table 2). The sequences of 8 *T. pleurotum*, and 13 *T. pleuroticola* strains, sourced from Hungary, Italy, Romania, Canada, USA, Netherlands, and Colombia, were used in order to determine the relationship of these strains and the isolates originating from Poland. ClustalW (Thompson et al. 1994) was used to align the sequences.

**Table 2:** The list of *Trichoderma* strains selected from the NCBI GeneBank database and used for the comparative analysis

Strain no.	Other collection	Origin	Habitat	NCBI GenBank accession no.	
				ITS	*tef1*
*T. pleurotum*					
C.P.K. 2113	CBS121147, DAOM 236051	Hungary	*P. ostreatus* substratum	EF392808	EF392773
C.P.K. 2096		Hungary	*P. ostreatus* substratum	EF392797	EF392770
C.P.K.2097		Hungary	*P. ostreatus* substratum	EF392798	EF392771
C.P.K. 2100		Hungary	*P. ostreatus* substratum	EF392801	EF392772
C.P.K. 2116	CBS 121148	Hungary	*P. ostreatus* substratum	EF392810	EF392774
C.P.K. 2117		Hungary	*P. ostreatus* substratum	EF392811	EF392775
C.P.K. 1532	CBS 121216	Italy	*P. ostreatus* substratum	EF392795	EF601678
C.P.K. 2815		Romania	*P. ostreatus* substratum	EF601675	EF601680
*T. pleuroticola*					
DAOM 175924	CBS121144	Canada	*Acer* sp.	AY605726	AY605769
DAOM 229916		USA	Forest soil	AY605738	AY605781
C.P.K. 1540	CBS 121217	Italy	*P. ostreatus* incubating bales	EF392782	EF392762
C.P.K. 1544		Italy	*P. ostreatus* incubating bales	EF392786	EF392763
C.P.K. 1550		Italy	Mushroom farm	EF392791	EF392765
C.P.K. 1551		Italy	Mushroom farm	EF392792	EF392766
C.P.K. 2104	CBS 121145	Hungary	*P. ostreatus* substratum	EF392794	EF392769
C.P.K. 3266		Hungary	*Populus Canadensis* stump	EU918148	EU918160
C.P.K. 3193		Hungary	*Populus alba* stump	EU918140	EU918160
C.P.K. 2816		Romania	*P. ostreatus* substratum	EF601676	EF601681
C.P.K. 2817		Romania	*P. ostreatus* substratum	EF601677	EF601682
G.J.S. 95-81		The Netherlands	*Pleurotus* spawn	AF345948	AF348102
T 1295		Colombia	Soil	EU280071.1	EU279973.1

## Results

### Identification of *T. pleurotum* and *T. pleuroticola* isolates

Preliminary identifications of the 80 *Trichoderma* isolates collected from the 47 oyster mushroom farms in Poland and 9 reference strains (E135, E136, E137, E138, E139, M141, M142, M143, M144) were based both on phenetic observations and multiplex PCR assay. PCR amplification with primers FPforw1, FPrev1, and PSrev1 expressed 447- and 218-bp fragments in 47 examined isolates and 4 reference strains (E135, E136, E138, E139), characterised as *T. pleurotum*. Only the larger band of 447 bp was observed in two examined isolates (T12/B, T24/T) and five references strains (E137, M141, M142, M143, M144) of *Trichoderma*. This indicated the presence of *T. pleuroticola*. However, no amplified product was detected in the remaining (31) *Trichoderma* isolates.

The initial identifications of 2 *T. pleuroticola* and 47 *T. pleurotum* isolates collected from Poland as well as 9 reference *Trichoderma* strains were confirmed by sequencing two different phylogenetic markers: the fragment of the ITS1-5.8S-ITS2 rRNA region and the fragment of the *tef1* gene (Table 1). The sequence analyses were also used to identify the remaining *Trichoderma* isolates collected from oyster mushroom farms in Poland. These isolates were identified as *Trichoderma harzianum Rifai* (17 isolates) and *Trichoderma atroviride* P. Karst (14 isolates) (Table 1).

### Comparison of ITS and *tef1* sequences of *T. pleurotum* and *T. pleuroticola* isolates

The comparative analyses were based on the ITS and *tef1* sequences of the *T. pleurotum* and *T. pleuroticola* strains both obtained in this study and published previously by Hatvani et al. (2007), Komon-Zelazowska et al. (2007), and Kredics et al. (2009).

**Fig. 1:**
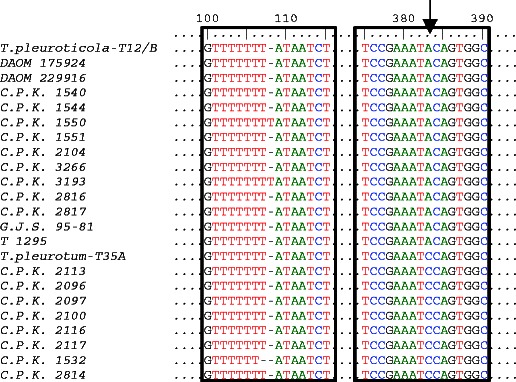
The intra- and interspecies variability in the ITS sequences of selected *T. pleurotum* and *T. pleuroticola* isolates from oyster mushroom farms in Poland and strains deposited in NCBI GeneBank (Tables 1 and 2). The nucleotide polymorphism for *T. pleurotum* and *T. pleuroticola* strains are enclosed. Single nucleotide polymorphism (A/C transversion) between ITS alleles of *T. pleurotum* and *T. pleuroticola* is shown by the *arrow*

DNA sequence alignment showed that the ITS allele detected in 47 *T. pleurotum* isolates from Poland was identical to that of *T. pleurotum* strains from Hungary (C.P.K. 2113, C.P.K. 2096, C.P.K. 2097, C.P.K. 2100, C.P.K. 2116, C.P.K. 2117) and Romania (C.P.K. 2814) but differed by one single nucleotide polymorphism (SNP) from the Italian strain C.P.K. 1532. Similarly, 2 *T. pleuroticola* isolates from Poland and 11 strains from: Canada (DAOM 175924), USA (DAOM 22996), Italy (C.P.K. 1540), Romania (C.P.K. 2816, C.P.K. 2817), Hungary (C.P.K. 2104, C.P.K. 3266), Netherlands (G.J.S. 95–81), and Colombia (T 1295) possessed an identical allele in the ITS locus, while their ITS1 sequences were different by one SNP from the sequences of Italian strain C.P.K. 1550 and Hungarian strain C.P.K. 3193. Single nucleotide polymorphism (A/C transversion) was also observed between ITS alleles of *T. pleurotum* and *T. pleuroticola* isolates used in the present study. The intra- and interspecies variability in the ITS sequences, deriving from single nucleotide indel or transition (A-C), is given in Fig. 1.

**Fig. 2:**
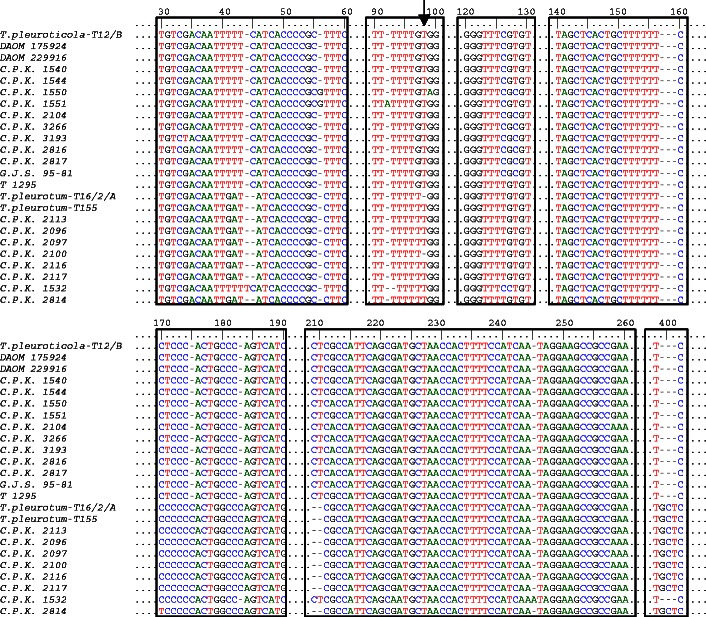
The intra- and interspecies variability in the *tef1* sequences of selected *T. pleurotum* and *T. pleuroticola* isolates from oyster mushroom farms in Poland and strains deposited in NCBI GeneBank (Tables 1 and 2). The nucleotide polymorphism for *T. pleurotum* and *T. pleuroticola* strains are enclosed. Single nucleotide insertion/deletion (“T”/“N” allele) between Polish *T. pleuroticola* isolates is shown by the *arrow*

As shown in Fig. 2, *T. pleurotum* and *T. pleuroticola* were clearly divergent in the *tef1* analysis. Their *tef1* sequences were separated by several indel and nucleotide substitutions. The set of 47 *T. pleurotum* isolates originating from Poland were found to be polymorphic and represented two *tef1* alleles (“T” type and “N” type), distinguishable based on one single nucleotide insertion/deletion (Fig. 2, Table 1). Nineteen Polish isolates of *T. pleurotum* possess the *tef1* allele (“T” type) identical to three isolates from Hungary (C.P.K. 2113, C.P.K. 2116, C.P.K. 2117) and Romania (C.P.K. 2814), but different from the alleles represented by Hungarian strain C.P.K. 2096, C.P.K. 2097, and C.P.K. 2100, and Italian strain C.P.K. 1532. The “N” type of the *tef1* allele, found in the remaining *T. pleurotum* isolates from Poland, has one position (indel or transition A/G) that differs from the allele type of five strains from Hungary (C.P.K. 2113, C.P.K. 2116, C.P.K. 2117, C.P.K. 2110) and Romania (C.P.K. 2814), two positions (indel and transition A/G) that differ from the allele type of two Hungarian strains C.P.K. 2096 and C.P.K. 2097, and several positions that differ from the allele type of Italian strain C.P.K. 1532. The *tef1* sequences of two *T. pleuroticola* isolates from Poland were identical to that of *T. pleuroticola* strains DAOM 175924 from Canada, DAOM 229916 from the USA, and C.P.K. 1540 and C.P.K. 1544 from Italy, but different by four A/G and T/C transitions from the sequences of C.P.K. 3266, C.P.K. 3193, C.P.K. 2816, C.P.K. 2817, and T 1295 strains. More polymorphism was detected between the *tef1* sequences of Polish *T. pleuroticola* isolates and that of C.P.K. 2104, C.P.K. 1550, and C.P.K. 1551 strains.

## Discussion

The present study states the association of *T. pleurotum* and *T. pleuroticola* with *Pleurotus* green mould in Polish mushroom farms. *T. pleurotum* was also the most common species collected from Hungarian oyster mushroom farms (Komon-Zelazowska et al. 2007). The predominance of *T. pleurotum* species in samples originating from Polish and Hungarian *Pleurotus* farms may be due to the use of similar technologies in the production of cereal straw substratum for mushroom cultivation. These technologies are different from the methods used in Italy (probably adverse for the *T. pleurotum* infection), where *T. pleuroticola* was the major contaminant of *Pleurotus* substratum (Komon-Zelazowska et al. 2007).

Other species isolated from green moulded substrata for *Pleurotus* cultivation in Poland were: *T. harzianum* and *T. atroviride*. The presence of these species in the cultivation of *P. ostreatus* was also noted by Hatvani et al. (2007). Additionally, Hatvani et al. (2007) found individual isolates of *Trichoderma longibrachiatum Rifai*, *Trichoderma ghanense Yoshim. Doi, Y. Abe & Sugiy*, and *Trichoderma asperellum Samuels, Lieckf. & Nirenberg*. Five of these seven species, namely *T. pleuroticola*, *T. harzianum*, *T. atroviride*, *T. longibrachiatum*, and *T. asperellum*, were isolated from the substrate and the basidiomes of wild-grown *P. ostreatus* in Hungary. *T. pleurotum* was not found in these samples.

The preliminary identification of the collected *Trichoderma* isolates was based on phenetic observations and multiplex PCR assay. DNA markers used in the present work and specific for *T. pleurotum* and *T. pleuroticola* were recently described by Kredics et al. (2009). These authors (Kredics et al. 2009) demonstrated that *T. pleurotum* and *T. pleuroticola* can be distinguished from each other, as well as from other fungal species, using three oligonucleotide primers: FPforw1, FPrev1, and PSrev1, based on *tef1* sequences. The present paper validates the specificity and the usefulness of the multiplex PCR assay developed by Kredics et al. (2009). As shown here, the PCR markers enabled the rapid screening of 80 *Trichoderma* isolates and specific detection of *T. pleurotum* and *T. pleuroticola*, collected from green moulded substrata for *Pleurotus* cultivation.

The ITS and *tef1* sequence information was used to establish the intra- and interspecies relationship of *T. pleurotum* and *T. pleuroticola* originating from the oyster mushroom farms in Poland and those from other countries. The comparative analysis of the ITS sequences showed that all *T. pleurotum* isolates from Poland represent one haplotype, identical to that of *T. pleurotum* strains C.P.K. 2113, C.P.K. 2096, C.P.K. 2097, C.P.K. 2100, C.P.K. 2116, C.P.K. 2117 from Hungary and C.P.K. 2814 from Romania, but different from Italian strain C.P.K. 1532. However, the sequence analysis of the *tef1* locus revealed two haplotypes of Polish *T. pleurotum* isolates—“T” type and “N” type. The “T” type isolates of *T. pleurotum* have identical *tef1* allele to that of strains C.P.K. 2113, C.P.K. 2116, C.P.K. 2117 from Hungary and C.P.K. 2814 from Romania, whereas the “N” type isolates are unique at the *tef1* locus. As observed in the present study, the distribution of “T” type and “N” type isolates in Poland is not correlated with the location of the mushroom farms from which they originated (Table 1). According to a previous study (Komon-Zelazowska et al. 2007), the source of *T. pleurotum* infection is the substratum for mushroom cultivation. Thus, the composition of two *T. pleurotum* haplotypes most likely depends on the manufacturer (source) of the cereal straw substratum used for the mushroom cultivation. The trading (import–export) of the *Pleurotus* substratum among European countries could also explain the identicalness of the “T” type isolates to the Hungarian and Romanian *T. pleurotum* strains. Interestingly, a similar mechanism of *T. aggressivum* distribution in *Agaricus* mushroom farms has been observed (Hatvani et al. 2007). It is noteworthy that *T. aggressivum*, just like *T. pleurotum*, has so far never been isolated from the natural environment. As observed in the previous studies, the major source of *T. aggressivum* infection was the compost and the origin of its constituents (Hatvani et al. 2007; Komon-Zelazowska et al. 2007). Hatvani et al. (2007) performed the comparison of two populations of *T. aggressivum* f. *europaeum* isolates from the British Islands and Hungary. The analysis of mtDNA showed that Hungarian isolates of *T. aggressivum* f. *europaeum* belong to the same population as the first isolates from Northern Ireland and England, while they all proved to be clearly different from *T. aggressivum* f. *aggressivum* isolates. Furthermore, the complete identity or low levels of variability of ITS1 and ITS2 sequences were also observed for *T. aggressivum* f. *europaeum* strains examined by Muthumeenakshi et al. (1998), Samuels et al. (2002), and Błaszczyk et al. (2011). These studies indicated that *T. aggressivum* f. *europaeum* strains most likely derived from the Western European epidemic lineage.

The detailed analysis of the ITS and *tef1* sequences showed that two *T. pleuroticola* isolates from Polish mushroom farms are identical to strains DAOM 175924 from Canada, DAOM 229916 from the USA, and C.P.K. 1540 and C.P.K 1544 from Italy, whereas they are different from the Hungarian and Romanian strains. It is known that *T. pleuroticola* occur in association with *P. ostreatus* growing in natural environments and in mushroom farms (Park et al. 2004a, b, 2006; Szekeres et al. 2005; Komon-Zelazowska et al. 2007; Kredics et al. 2009). This is why the sources of *T. pleuroticola* infection may be various (Kredics et al. 2009). A study of the vectors for *T. pleuroticola* into mushroom farms could explain the distribution of this pathogenic species in Polish mushroom farms. This need is highlighted by the present paper and previous work (Komon-Zelazowska et al. 2007).
